# Biological larviciding against malaria vector mosquitoes with *Bacillus thuringiensis israelensis (Bti)* – Long term observations and assessment of repeatability during an additional intervention year of a large-scale field trial in rural Burkina Faso

**DOI:** 10.1080/16549716.2020.1829828

**Published:** 2020-10-08

**Authors:** Peter Dambach, Volker Winkler, Till Bärnighausen, Issouf Traoré, Saidou Ouedraogo, Ali Sié, Rainer Sauerborn, Norbert Becker, Valérie R. Louis

**Affiliations:** a Institute of Global Health, University Hospital Heidelberg, Heidelberg, Germany; b Centre de Recherche en Santé de Nouna, Nouna, Burkina Faso; c German Mosquito Control Association (KABS), Speyer, Germany

**Keywords:** Biological vector control, sub-Saharan Africa, malaria control, large scale intervention trial

## Abstract

The first line of malaria vector control to date mainly relies on the use of long-lasting insecticidal nets (LLINs) and indoor residual spraying (IRS). For integrated vector management, targeting the vector larvae with biological larvicides such as *Bacillus thuringiensis israelensis* (*Bti*) can be an effective additional mainstay. This study presents data from the second intervention year of a large-scale trial on biological larviciding with *Bti* that was carried out in 127 rural villages and a semi-urban town in Burkina Faso. Here we present the reductions in malaria mosquitoes that were achieved by continuing the initial interventions for an additional year, important to assess sustainability and repeatability of the results from the first intervention year. Larviciding was performed applying two different larviciding choices ((a) treatment of all environmental breeding sites, and (b) selective treatment of those that were most productive for *Anopheles* larvae indicated by remote sensing based risk maps). Adult *Anopheles* spp. mosquito abundance was reduced by 77.4% (full treatment) and 63.5% (guided treatment) compared to the baseline year. The results showed that malaria vector abundance can be dramatically reduced using biological larviciding and that this effect can be achieved and maintained over several consecutive transmission seasons.

## Background

Impregnated bed nets and indoor residual spraying are the mainstays of malaria vector control, and they have strongly contributed to the considerable results achieved in malaria reduction worldwide [1]. However, the development of resistance to pyrethroids is decelerating and sometimes reversing current gains in malaria control [2,3]. Equally, shifts in vector biting behavior from night biting to early evening or early morning biting have been observed, evading bed net barriers [4–7]. An alternative approach to circumnavigate these limitations is to target the larval stages of mosquitoes in their breeding sites where they are concentrated, bounded and easily accessible. This larval source management comprises the elimination, transformation and treatment of larval breeding sites. Compared to early undertakings in the era of Dichlorodiphenyltrichloroethane (DDT), today´s vector control can make use of environmentally sound larvicides that cause no harm for humans or animals, including other insects. Field trials with the biological larvicide *Bacillus thuringiensis israelensis* (*Bti*) have shown the efficacy of reducing larvae and vector populations [8–11].

To research the feasibility and the impact of biological larviciding against malaria in a rural area of sub-Saharan Africa, we implemented a large-scale field trial, covering 127 rural villages and a semi-urban town in North-Western Burkina Faso. The study´s impact evaluation comprised several indicators on the epidemiological pathway, from mosquito larvae to human malaria infections [12]. This manuscript presents the mosquito reductions that were achieved during the second intervention year of the trial; the reductions of the first intervention year were presented elsewhere [11]. Performing the same larviciding interventions during an additional year, allowed us to show the repeatability of results achieved during the first intervention year, laying the foundation to better assess those interventions for their use in routine malaria control.

## Methods

The study was conducted in the Kossi region in North-Western Burkina Faso and covered all 127 rural villages of the Nouna health district and the semi-urban town of Nouna itself. The region is subject to year-round malaria transmission with a distinct maximum during the rainy season, which extends from beginning of July through beginning of October. The principal malaria vectors are *Anopheles gambiae* sensu lato, making up more than 90% of the population, followed by *A. funestus* and *A. nili* [5].

The study setup comprised three arms with different larviciding choices: exhaustive treatment of all breeding sites (full treatment), guided treatment of only the breeding sites with the highest larval densities determined by remote sensing-based risk maps, and an untreated control group (described in detail elsewhere [12]). As in the first treatment year 2014, in 2015 larviciding was performed with *Bti* VectoBac® WG, AM65-52 strain (Valent BioSciences Corporation, IL, USA) during and after the rainy season from July throughout October. The 2015 season focused on indoor mosquitoes because our earlier results indicated greater mosquito abundance indoors (34.0% more on average). Mosquito captures were carried out using Center for Disease Control light traps (Model 512, John W. Hock Company, Gainesville, Florida) in 36 villages and seven town quarters of the district capital. To cover all geographical areas, mosquito sampling rounds (called ‘batches’) were performed over two-week periods. At least 10 sample rounds per village took place during each annual rainy season.

Statistical analysis was performed using Stata/IC 14.2 for Windows (StataCorp LLC, 4905 Lakeway Drive, College Station, TX 77845, USA). A Poisson regression was performed to model the number of female *Anopheles* spp. mosquito counts per trap per night using the following categorical variables: batch, treatment choice, year, and the interaction term (difference-in-difference estimates) between treatment choice and year. Standard errors allowed for intragroup correlation at the village level.

## Results

**Figure 1.**
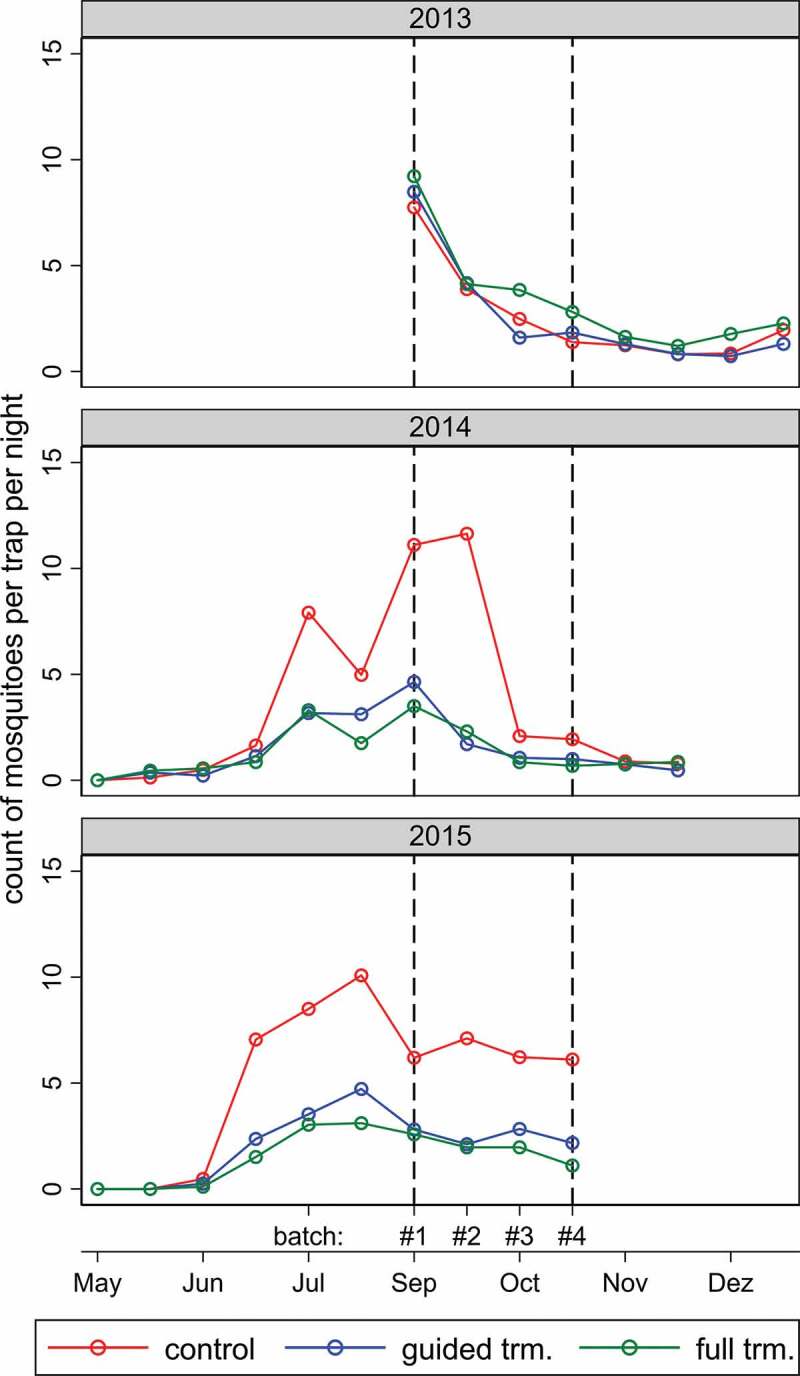
Average numbers of female Anopheles mosquitoes per trap per night captured indoors during successive sampling rounds of the three study years. The colors correspond to the average values in geographical areas receiving different Bti treatments in 2014 and 2015 (2013 was the baseline year). The vertical dotted lines indicate the common sampling period over the 3 years. Trm = treatment.

Mosquito abundance varied over time (Figure 1). Results from the baseline year (2013, no *Bti* treatment) indicate that mosquito abundance exhibited a similar pattern over time in each geographical area corresponding to a specific treatment arm. In 2013, mosquito abundance was comparable in all three treatment zones but slightly elevated in the full treatment area. As expected, mosquito abundance was reduced in treatment areas during treatment years (2014 and 2015). In the control areas during the rainy season (July-October), the natural mosquito abundance in 2015 was on average 24.3% higher compared to 2014, and in 68.8% of the villages the mosquito counts were higher in 2015 compared to 2014. As a result, the same reduction rates resulted in higher absolute numbers of *Anopheles* that were captured per trap per night. The September and October mosquito counts per night per trap in the control villages were higher in 2015 with on average 6.4 ± 2.0 female *Anopheles* compared to with 3.8 ± 2.5 in the baseline year. In 2015, spatial variability was highest among control villages with a standard deviation ranging from ± 0.56 to ± 3.42 mosquitoes per night per trap. Unsurprisingly, the variations were lower in treated areas (guided treatment: ± 0.34 to ± 1.26; full treatment: ± 0.18 to ± 1.20) because the lower number of mosquitoes captured reduced the variability.

**Figure 2.**
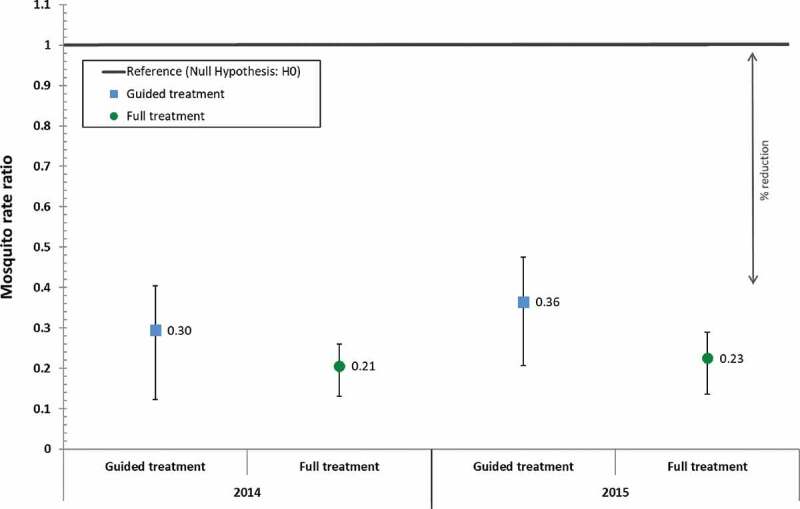
Difference-in-difference estimates during the common sampling period obtained with a Poisson regression model comparing the intervention years with the baseline year and indicating the reduction in the count of indoor female Anopheles mosquitoes per night per trap achieved through guided or full Bti treatment. The reference line represents the rate ratio value of 1 under the null hypothesis. (p-values were <0.001 for all entries).

Statistical analysis of data during the common sampling period (Figure 2, Table S1) shows that larviciding with *Bti* reduced the *Anopheles* densities at indoor capture posts in 2015 by 77.4% (95% CI: 68.4% – 83.8%) in the full treatment arm and by 63.5% (95% CI: 47.8% – 74.5%) in the guided treatment arm. These reductions were slightly lower but comparable to those achieved at indoor capture posts in the previous year (full: 79.4% [95% CI: 71.9% – 84.9%]; guided: 70.5% [95% CI: 53.3% – 81.3%]).

## Discussion

We showed that malaria vector abundance in the second intervention year was largely reduced, and that attained rate ratios were comparable to those of the preceding intervention year 2014 for both treatment choices [11]. The estimated reduction with the difference-in-difference approach took into account the natural mosquito increase, which was observed in the control areas. The second intervention year featured higher natural *Anopheles* spp. abundance. This indicates, that similar reduction rates in adult vector mosquitoes are likely to be achievable over extended periods of time, even through years with naturally higher mosquito infestations. These adult vector reductions were realized at moderate yearly per capita intervention costs of US$ 1.05 for the full treatment and US$ 0.77 for the guided treatment, despite the rural nature of the study villages. The spatial variability of mosquito reductions among villages indicates that the assessment of the efficacy of larviciding interventions needs to be based on a mixed calculation of a larger area. The reductions achieved in 2015 and in the previous year are comparable to findings from Kenya [13] where reductions of 85.9% have been observed during indoor resting collections. Other studies found reductions of more than 80% in the Entomological Inoculation Rates, while reductions at resting stations were lower [8].

Although we limited the mosquito collections to indoor sample points, our results underline that the impact of biological larviciding on *Anopheles* vector populations is reproducible and was almost identical between our two intervention years. Having this data available from a large-scale trial over the period of two years, is valuable and might help to better estimate the entomological impact and sustainability of such interventions in a rural African environment over longer periods of time, which is important for assessing their usefulness as a routine measure.

## Supplementary Material

Supplemental Material

## Paper context

Biological larviciding is a method of controlling mosquito larvae in their breeding sites. Although the approach has been shown effective in several trials in urban settings, evidence of the feasibility and effectiveness of a large-scale intervention in a rural African environment had not yet been provided. With this trial we tried to address this lack of evidence, and additionally, to evaluate if remote sensing derived risk maps can further improve efficient use of the larvicide.
