# Supplementary material for: Biological larviciding against malaria vector mosquitoes with Bacillus thuringiensis israelensis (Bti) – Long term observations and assessment of repeatability during an additional intervention year of a large-scale field trial in rural Burkina Faso
Source: Glob Health Action. 2020 Oct 8;13(1):1829828. doi: 10.1080/16549716.2020.1829828 (PMC7580761; doi:10.1080/16549716.2020.1829828)
Supplement: Supplementary file 1 [file ZGHA_A_1829828_SM8608.odt]

Table S1: Poisson regression estimating the number of female Anopheles mosquito counts per trap per night with random effect for village level

|  | Estimate | 95% CI | p-value |
| --- | --- | --- | --- |
| Treatment choice |  |  | <0.001 |
| control (reference) | 0 | - |  |
| guided trm. | 0.058 | -0.288 – 0.405 |  |
| full trm. | 0.273 | 0.031 – 0.516 |  |
| Year |  |  | <0.001 |
| 2013 (reference) | 0 | - |  |
| 2014 | 0.566 | 0.340 – 0.792 |  |
| 2015 | 0.512 | 0.258 – 0.766 |  |
| Treatment × Year |  |  |  |
| reference | 0 | - | <0.001 |
| guided trm. 2014 | -1.220 | -1.679 – -0.762 |  |
| guided trm. 2015 | -1.008 | -1.367 – -0.649 |  |
| full trm. 2014 | -1.580 | -1.890 – -1.270 |  |
| full trm. 2015 | -1.487 | -1.822 – -1.153 |  |
| Batch |  |  | <0.001 |
| batch 1 | 0 | - |  |
| batch 2 | -0.348 | -0.487 – -0.209 |  |
| batch 3 | -0.854 | -1.000 – -0.708 |  |
| batch 4 | -1.050 | -1.201 – -0.899 |  |
| Constant | 1.823 | 1.606 – 2.040 | <0.001 |
